# The Werther Effect of Two Celebrity Suicides: an Entertainer and a Politician

**DOI:** 10.1371/journal.pone.0084876

**Published:** 2013-12-26

**Authors:** Jae-Hyun Kim, Eun-Cheol Park, Jung-Mo Nam, SoHee Park, Jaelim Cho, Sun-Jung Kim, Jae-Woo Choi, Eun Cho

**Affiliations:** 1 Department of Public Health, Yonsei University, Seoul, Republic of Korea; 2 Institute of Health Services Research, Yonsei University, Seoul, Republic of Korea; 3 Department of Preventive Medicine, Yonsei University College of Medicine, Seoul, Republic of Korea; 4 School of Public Health, Yonsei University, Seoul, Republic of Korea; 5 College of Pharmacy, Sookmyung Women’s University, Seoul, Republic of Korea; Durham University, United Kingdom

## Abstract

**Purpose:**

Suicide is a major health problem in Korea. Extensive media exposure of celebrity suicide may induce imitative suicide, a phenomenon called the Werther effect. We examined the increased suicide risk following the suicides of an entertainer and a politician, and identified the relative suicide risks.

**Methods:**

News articles about the celebrity suicides were obtained from three major newspapers and analysed for quantitative and qualitative features. Imitative suicide risk was investigated by applying a Poisson time series autoregression model with suicide mortality data from the National Statistics Office for 1.5 years before and 1.5 years after each celebrity’s suicide. The period with a significantly increased number of suicides immediately after the celebrity’s suicide determined the Werther effect band. The relative risk during this period was examined for different ages, genders, and suicide methods.

**Results:**

News reports were more numerous and they contained more positive definitions about the entertainer’s suicide. The risk of suicide deaths rose markedly after both celebrity suicides. However, the Werther effect band was longer for the entertainer (6 weeks) than for the politician (4 weeks). The relative suicide risk was significant for almost all ages and both genders during that of both individuals. Use of the same suicide method was a prominent risk factor after both celebrity suicides.

**Conclusions:**

Our results confirm the existence of imitative suicide behaviours, suggesting a facilitation effect of media reports. Guidelines for responsible media reporting need to be implemented to enhance public mental health in Korea.

## Introduction

Suicide is a major health issue in all countries[1]. According to the Korean National Statistics Office, suicide-caused death has increased by 119.90% during the last decade. In 2011, 43.6 people died by suicide daily in Korea[2]. The suicide rate of 31.7 per 100,000 people is about 2.6 times higher than the OECD average; thus, suicide is a serious social issue in Korea[2]. The suicide rate is particularly high among elderly people, reflecting the rapid aging of Korean society and the suicide rate in males outnumbers females as in most other parts of the world[2-4]. 

Celebrity suicides have frequently been reported in the media over the last decade in Korea. A celebrity suicide event is likely to receive public attention through extensive media exposure, and this event may induce imitative suicides[5-7]. The surge in the incidence of suicide and suicide attempts following incidents of celebrity suicide is particularly salient when those celebrity suicide cases are widely publicised in the media[8-12]. This general imitative effect of an increase in the number of suicides triggered by extensive media reporting on suicide cases is described as the ‘‘Werther effect’’, which describes the widespread imitative suicide copying Werther (the hero in Goethe’s novel)[12]. Since the term Werther effect was used to describe the phenomenon of the increasing suicides after a publicized suicide in a 1974 article, it has been widely discussed in terms of the impact and mechanisms of publicized report on suicide behaviour[12-15]. Previous studies have suggested that age, gender and identification of the method of suicide following a celebrity suicide are relevant to imitative suicide[8-10,12,16,17]. This imitative suicide is more prominent among young people and among the same gender as the celebrity[5,9,11,17-19]. 

Since the Werther effect was reported in Taiwan[10,11,18], further research estimated the elevated suicide risk after celebrity suicides in non-western countries[8,9,17,20]. Regarding Korea where the suicide rate is increasing rapidly, one study reported an increased suicide risk in Korea after the suicide death of a famous actress in 2005[17]. Since then, subsequent suicide deaths of celebrities have occurred in Korea. A recent study examined a pattern of increasing suicide rates in Korea and found that three of 11 suicides of celebrities significantly influenced the suicide risk in the country[20]. While media effects about celebrities’ suicide and imitative suicides are linked, given their renown[21], the Werther effect after the suicide of other types of celebrity, rather than an entertainment celebrity, has rarely been examined. In a milestone study investigating on the celebrity suicide effects based on the theoretical taxonomy of celebrities, only entertainers and political celebrities were found to be related to significant Werther effect among several types of celebrity[21].

During the last decade, two dramatic examples of celebrity suicide in Korea were the deaths of actress Jin-Sil Choi and the former President Moo-hyun Roh of the Republic of Korea. A popular 40-year-old movie actress, Jin-Sil Choi, killed herself by hanging in her house on October 2, 2008. The next year, the 63-year-old sixteenth Korean President, Moo-hyun Roh, killed himself by jumping off a mountain on May 23, 2009. Ms. Choi was a major star who overcame childhood poverty and difficulties. Giving hope to the public, she was popularly called ‘the nation’s female actor’. Former President Roh, who used to represent politically disadvantaged people as a civil-rights lawyer in South Korea, was elected as the sixteenth Korean President (February 25, 2003-February 24, 2008). He received extensive support, particularly from the younger population. Before their suicides, these two celebrities were living in a state of considerable distress. The distress and suffering of Ms. Choi had been continuously reported following her divorce from her husband, due to his infidelity, approximately 3 years before her suicide. Sympathy for her tragedy went hand-in-hand with reproach of her former husband immediately after her suicide, which was amplified by a number of imitative suicides. Towards the end of his tenure, President Roh suffered from depression and was under pressure from the opposing party, who brought about a regime change, regarding evidence of his political corruption, which constituted a fatal blow to his image as an ‘ordinary person’ who differed from the public perception of typical politicians[22]. The disappointment of the general population in President Roh may have weakened the Werther effect following his death, as compared to that of Ms. Choi.

News of the suicides appeared immediately in the headlines of TV networks, newspapers, and Internet broadcast. The methods of the suicides were detailed and pictures of the suicide scenes were aired for several weeks. 

In this study, we further examined the effect of politician celebrity and entertainment celebrity suicide news on the suicide rate across age, gender, and suicide method. We also investigated the duration of the Werther effect in terms of the age, sex and suicide method of suicides that may have resulted from the media reporting of the suicides of an entertainment celebrity and a political celebrity. 

## Materials and Methods

### Media reporting

We selected the three most widely circulated newspapers in Korea to identify the numbers of news reports and to examine the qualitative features of news articles about the two celebrity suicides. During the 3 weeks after the suicide of Ms. Choi, 905 news articles focusing on her suicide were identified. The suicide of former President Roh was reported in 360 news articles during the same period after the suicide. The majority of the news reports appeared in the first week after each celebrity death (Ms. Choi, n = 792, 87.5%; President Roh, n = 331, 91.9%). 

In the news articles, the celebrity’s name and picture, suicide method and the picture of the suicide site were provided in detail for both suicides. However, some qualitative features differed between the news content delivered for the two suicides. For example, the private affairs of Ms. Choi in public life, such as her divorce from a famous sports star, were already widely known to the general public a few years before her suicide. Along with the suicide news, the present condition and pictures of her divorced former husband, close celebrity friends, and bereaved family were intensively reported for 3 weeks. Because of corruption scandals implicating individuals including relatives and his aides during his tenure (February 2003-February 2008), former President Roh was also under a prosecutory investigation at that time. However, the names of, and reports on, individuals related to his infidelity were seldom mentioned in those news articles. 

According to the news frame classification[23], a ‘cherishing’ frame was predominant in the news reports of Ms. Choi’s suicide, whereas a conflicted view involving a cherishing frame and a personal misery frame coexisted in news reports about President Roh. Furthermore, regarding the responsibility for former President Roh’s suicide, different arguments were also present; some saw the prosecution as responsible while others saw the need for continuing investigation. 

### Suicide and time-series data

Official suicide mortality data were obtained from the National Statistics Office for 2007–2010 in Korea[24]. The code for suicide is X600-X849 in the Korean Classification of Diseases, Sixth Revision, based on the International Classification of Diseases[25]. Hanging is coded as X700-X709 and jumping is coded as X800-X809. 

Since environmental factors might be related to suicidal risk, weather variables such as average temperature and humidity were adjusted as confounding effects in our time-series analysis as well as seasonal variation. Chronological records of average temperature and humidity were retrieved from the Korea Meteorological Administration[26]. As a secular confounding factor, unemployment rate was also considered in our analysis. The monthly unemployment rate data were obtained from the Web site of the National Statistical Office of Korea on the risk of suicide. The weekly unemployment rate was estimated by the rate for the corresponding month period.

### Statistical analyses

A Poisson time-series autoregression model was constructed for the two celebrities for the 1.5 years before and the 1.5 years after the suicide of each to examine changes in suicides. The weekly suicide counts from May 2007 to October 2010 were treated as a Poisson distribution. Thus, the relative risk (RR) of suicide was estimated by the number of suicide events that occurred after the celebrity suicide event divided by the number of suicides in the remaining weeks between May 2007 and March 2009 for Ms. Choi’s suicide case and between December 2007 and October 2010 for former President Roh. 

The Werther effect band was examined as the period with a statistically significantly increased suicide risk immediately after the celebrity’s suicide event. The elevated suicide risk during the Werther effect band over the other time period during the 3 years was also investigated by applying a Poisson autoregressive model according to age group, gender, method of suicide (same suicide method used by the respective celebrity or other method) and year. 

The uniqueness of the Werther effect band was examined by establishing a Poisson time-series autoregression model on the same period of Werther effect band for one calendar year before and one year after celebrity suicide year. In addition, the risk of using the specific suicide method with celebrity in the same period of Werther effect band during each calendar year before and after celebrity suicide year was analysed to prove the validity for the elevated risk during the Werther effect. 

Time-related variables including season, day of the week, calendar year, and time period were treated as categorical variables, while average temperature, humidity, and unemployment rate were treated as continuous variables in our model.

## Results

### Suicide rates after the celebrity suicides

**Figure 1:**
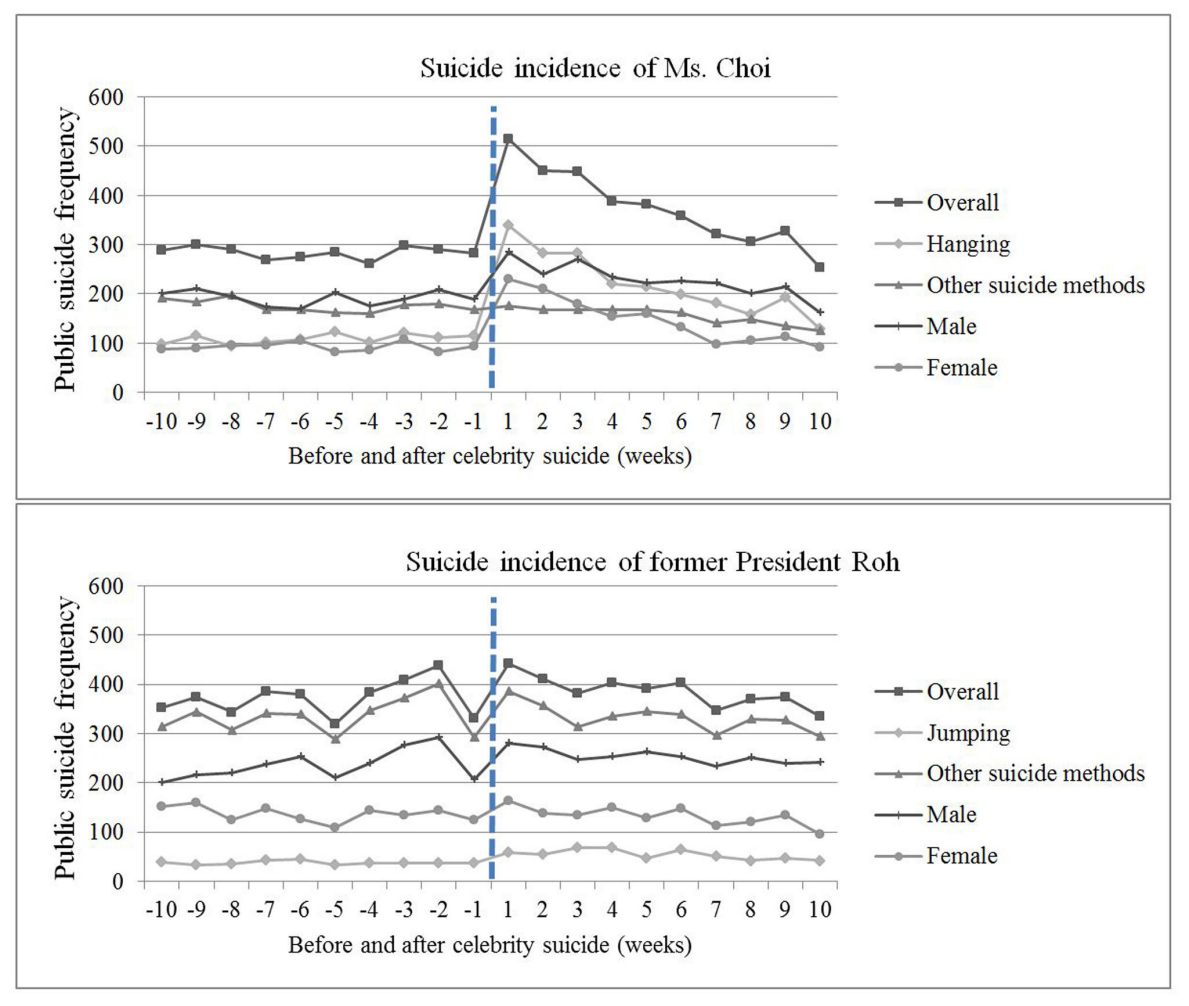
Weekly number of suicides before and after the celebrity suicides.

The suicide incidence rate before and after each celebrity suicide on a weekly basis is depicted in Figure 1. Immediately after both suicides, the suicide rate rose. However, the increase in the suicide rate appeared steeper during the first week after the suicide relative to the week before in Ms. Choi’s case (515 vs. 283 suicides; 1.82 times) as compared to the increase after that of President Roh (442 vs. 331 suicides; 1.34 times). Subsequently, suicides gradually decreased. During the second and third weeks after Ms. Choi’s suicide, there were 451 and 449 suicides. Comparing the incidence of suicide in the 3-week periods before and after the suicide of the celebrity, there were increases of 162.3% and 104.8% following Ms. Choi’s and President Roh’s suicides, respectively (Table 1). 

**Table 1:** Number of suicides in the 3-week period prior to, and after, the suicide of actress Choi and former President Roh in South Korea.

	Three weeks before the suicide	Three weeks after the suicide	% change of increase	P-value
Actress Choi (2008)				
All	872	1415	62.27%	<.0001
Male	588	795	35.20%	<.0001
Female	284	620	118.30%	<.0001
<50 years	431	799	85.38%	<.0001
By hanging	588	775	31.80%	<.0001
Ex-President Roh (2009)				
All	1179	1236	4.83%	0.4025
Male	776	800	3.09%	0.6516
Female	403	436	8.19%	0.2929
>=50 years	605	645	6.61%	0.3485
By jumping	112	180	60.71%	0.0003

P-value is associated with t-test.

While suicide rates rose significantly for both males and females during the 3 weeks after Ms. Choi’s death, the female suicide rate more than doubled (118.3% vs. 35.2%) (Table 1). The increased rates of the male and female groups were not statistically significant in President Roh’s case (Figure 1, Table 1). Suicides by subjects < 50 years of age increased significantly by 85.38% during the 3 weeks immediately after Ms. Choi’s suicide (p < 0.0001), whereas immediately after former President Roh’s suicide the increase was only 6.61% for subjects over 50 years of age (Table 1). 

Hanging suicides, which was the method used by Ms. Choi, increased significantly (60.71% increase; p < 0.0001). The number of suicides by jumping, which was the suicide method used by President Roh, also increased markedly to 180 (60.71%) from 112 (p = 0.0003). 

### Adjusted RR of suicide

The estimates of our generalised additive model for the adjusted suicide risk over 2 years with a celebrity’s suicide as the mid-point are presented in Table 2. After adjusting for other factors, spring, summer and fall were all significantly associated with an increased number of suicides regarding both Ms. Choi’s and former President Roh’s suicide event models. 

**Table 2:** Relative risk for factors associated with suicides during the 3 years with celebrity suicide as the center.

		Actress Jin-Sil Choi (40 years old in Oct 2008)		Ex-president Moo-hyun Roh (63 years old in May 2009)	
Factors		Adjusted Relative Risk	95% Confidence interval	Adjusted Relative Risk	95% Confidence interval
Season	Winter	1.000		1.000	1.000
	Spring	1.203^*^	1.152 – 1.256	1.153^*^	1.102 – 1.207
	Summer	1.191^*^	1.124 – 1.263	1.094^*^	1.027 – 1.165
	Fall	1.150^*^	1.095 – 1.208	1.180^*^	1.122 – 1.242
Days of the week	Sun	1.000			
	Mon	1.139^*^	1.089 – 1.191	1.139^*^	1.086 – 1.194
	Tue	1.106^*^	1.058 – 1.157	1.105^*^	1.054 – 1.159
	Wed	1.059^*^	1.012 – 1.108	1.057^*^	1.008 – 1.109
	Thu	1.043	0.997 – 1.092	1.041	0.992 – 1.093
	Fri	1.030	0.984 – 1.078	1.029	0.980 – 1.080
	Sat	0.982	0.938 – 1.029	0.981	0.934 – 1.031
Humidity		0.998^*^	0.997 – 0.999	0.998^*^	0.996 – 0.999
Temperature		1.008^*^	1.005 – 1.010	1.010^*^	1.008 – 1.013
Unemployment rates		1.101	1.052 – 1.152	1.084	1.032 – 1.464
Time-Day		1.000^*^	1.000 – 1.000	1.000^*^	1.000 – 1.000
Time-Week	Other period**^*a*^**	1.000		1.000	
	1st week after suicide	1.726^*^	1.537 – 1.939	1.283^*^	1.124 – 1.464
	2nd week	1.518^*^	1.342 – 1.717	1.237^*^	1.080 – 1.417
	3rd week	1.513^*^	1.338 – 1.712	1.168^*^	1.014 – 1.347
	4th week	1.316^*^	1.153 – 1.501	1.212^*^	1.055 – 1.392
	5th week	1.297^*^	1.136 – 1.481	1.144	0.994 – 1.317
	6th week	1.240^*^	1.082 – 1.421	1.218^*^	1.061 – 1.398
	7th week	1.115	0.065 – 1.288	1.058	0.912 – 1.226

Poisson time series autoregression model was applied for the 3-year period (1.5 years before and 1.5 years after the celebrity’s suicide).

^*^ P< 0.05.

^a^ Remaining weeks apart from the first 7 weeks after celebrity suicide

Among the days of the week, Monday through Wednesday had significantly elevated suicide risk, as compared to Sunday, for both Ms. Choi and President Roh’s cases (Table 2). Humidity was significantly associated with suicide risk for the periods of the two celebrity suicides. As the temperature rose one degree Celsius, the relative suicide risk increased by 1–1.8% after adjusting for potential confounders in the time series model, which was significant for both Ms. Choi’s and President Roh’s suicide periods. The influence of unemployment rate on suicide risk was not statistically significant (Table 2).

When we looked at the incidence of suicides on a weekly basis in the 7 weeks immediately after the celebrity suicide, there were significantly increased numbers of suicides from the first week to the sixth week following Ms. Choi suicide as compared to the period apart from the 7 weeks after adjusting for potential confounding factors (Table 2). Therefore, the Werther effect band was determined to be 6 weeks for the suicide of Ms. Choi. Following the suicide of former President Roh, the significantly increased adjusted RR lasted for 4 weeks and thus, the Werther effect caused by the suicide of President Roh persisted for 4 weeks (Table 2). 

The adjusted RR of factors during the Werther effect band is presented in Table 3 for seven different age category subgroups, gender, and suicide method during the 3 years. Apart from suicides by those aged < 20 years, all other age groups showed a significantly increased suicide risk in the Werther effect band of Ms. Choi. In particular, the adjusted RR during the Werther effect was greater by as much as 2.082 and 1.662 for suicides by subjects in their 20s and 30s (Table 3). After adjusting for all environmental and secular factors, the RR during the Werther effect band following the death of President Roh increased for suicides of all age groups with statistical significance as compared to the period outside the Werther effect band (Table 3). 

**Table 3:** Relative suicide risk of subgroups for age, gender and suicide method for suicides that occurred within the Werther effect band, as compared to other suicides at other times over 3 years.

		Actress Jin-Sil Choi (Werther effect band: 6 weeks)		Ex-president Moo-hyun Roh (Werther effect band: 4 weeks)	
Subgroup		Adjusted Relative Risk**^*a*^**	95% Confidence interval	Adjusted Relative Risk**^*b*^**	95% Confidence interval
Age	≤19	1.145	0.864 – 1.518	1.627^*^	1.117 – 2.368
	20-29	2.082^*^	1.819 – 2.382	1.664^*^	1.226 – 2.258
	30-39	1.662^*^	1.474 – 1.874	1.483^*^	1.120 – 1.964
	40-49	1.457^*^	1.301 – 1.632	1.573^*^	1.211 – 2.043
	50-59	1.224^*^	1.079 – 1.388	1.607^*^	1.243 – 2.078
	60-69	1.223^*^	1.074 – 1.393	1.457^*^	1.112 – 1.909
	≥70	1.117^*^	0.998 – 1.249	1.396^*^	1.099 – 1.772
Gender	Male	1.225^*^	1.143 – 1.312	1.523^*^	1.209 – 1.918
	Female	1.732^*^	1.592 – 1.885	1.519^*^	1.182 – 1.952
Method of suicide	Same suicide method	1.887^*^	1.741 – 2.045	1.537^*^	1.204 – 1.961
	Others	1.020	0.861 – 1.208	1.506	1.195 – 1.896
Same period of Werther effect band**^*c*^**	The year before suicide (2007 for Choi; 2008 for Roh)	1.030	0.951 – 1.117	1.023	0.930 – 1.125
	The year after suicide (2009 for Choi; 2010 for Roh)	0.994	0.921 – 1.074	0.949	0.881 – 1.023
Same suicide method during the same period of Werther effect**^*c*^**	The year before suicide (2007 for Choi; 2008 for Roh)	1.014	0.901 – 1.141	1.003	0.845 – 1.191
	The year after suicide (2009 for Choi; 2010 for Roh)	0.958	0.863 – 1.064	0.890^*^	0.797 – 0.994

Estimates were adjusted for the effects of season, day of the week, humidity, temperature, time of day, and unemployment rate.

^*^ P < 0.05.

^a^ The reference group was that of the suicides that occurred beyond the Werther effect band (6 weeks right after Choi’s suicide, October 2008) over three years (1.5 years before and 1.5 years after the suicide of actress Choi)

^b^ The reference group was that of the suicides that occurred beyond the Werther effect band (4 weeks right after former President Roh’s suicide, May 2009) over three years (1.5 years before and 1.5 years after the suicide of former President Roh).

^c^ The adjusted relative risk ratios during the same period of Werther effect band were measured for each of three calendar years: one year before; one year of; and one year after the suicide incident year.

The risk increased significantly during the Werther effect band of Ms. Choi for both male (adjusted RR = 1.225) and female suicides (adjusted RR = 1.732). The adjusted relative suicide risk for male suicides was considerably elevated (adjusted RR = 1.523) as well as for female suicides (adjusted RR = 1.519) during the Werther effect band period after former President Roh`s suicide (Table 3). 

Suicides using the same suicide method (hanging) notably increased (adjusted RR = 1.887) during the Werther effect band of Ms. Choi; other suicide methods also tended to increase, but not significantly so. Similar to the case of Ms. Choi, the suicide risk using the same suicide method (jumping) increased significantly (adjusted RR = 1.537) during the Werther effect band of President Roh, as compared to other times during the year before and the year after his suicide (Table 3). 

The elevated suicide risk during the same period of Werther effect (6 weeks immediately after October 2) was not observed in 2007 and 2009, the adjacent calendar years of the suicide death year for Ms. Choi’s case (Table 3). Likewise, for the 4-week (immediately after May 23) period of Werther effect, the significant difference in suicide death rate was not detected compared to other time period during the year before and during the year after the suicide year of former President Roh (Table 3). 

The adjusted RRs of method-specific suicide rate during the same period with the determined Werther effect band were not significantly different or rather reduced compared to the other time period during each of the previous year of and the next year of the suicide incident year for both Ms. Choi’s and former President Roh (Table 3). 

## Discussion

We have provided data about imitative behaviours in terms of the Werther effect following two celebrity suicides, one a popular actress and one a former President, over 3 years. These suicides were highly sensational and shocking incidents in Korea, where suicide is a major public health problem. A recently published study on the entertainer celebrity suicides in Korea showed that only three incidents among 11 were significant in terms of the impact on the suicide risk and Ms. Choi’s suicide case resulted in the strongest immediate impact on suicide deaths[20]. 

Although entertainers and politicians are very different types of celebrity, we found that both celebrities had a profound impact on suicide risk in terms of the Werther effect following their suicides. While the lives of both Ms. Choi and the former President influenced the public’s emotion, our research showed that the Werther effect band was a bit longer following the suicide of Ms. Choi (6 weeks) than after that of President Roh (4 weeks). Possible reasons for this discrepancy might be that the emotional connection of the public may be greater for an entertainer than for a politician, that President Roh’s suicide had less effect because it was less widely reported, and that the differences in the news frame used in relation to these two celebrity suicides may have affected the duration of the Werther effect band. Considering the longer period of Werther effect band for Ms. Choi’s incident could be explained by the fact of greater media coverage for Ms. Choi’s suicide in terms of the counts of newspaper columns for 3 weeks (n = 905) compared to former President Roh (n = 360). Stack’s work supports our results as that it proved that the greater the media coverage measured in column inches, the greater the suicide risk and this imitative suicide was triggered only for entertainer and politician celebrity subtypes[21]. 

Conflicting news frames in newspaper articles on the suicide of President Roh indicate a potentially weakened empathy toward him. In addition, the media reporting on the suicide of former President Roh may have been more limited by social norms and, hence, his suicide was less of a shock to the public. According to differential identification theory, negative definitions of suicide through negative frame of media coverage weakened the imitative suicide behaviours[27].

While previous findings have shown that young people are more susceptible to the influence of celebrity suicides[6], and that being middle-aged is a protective factor against media influence[28], our findings reveal a significantly higher suicide risk among all age groups during the Werther effect band for both Ms. Choi and President Roh. 

While the Werther effect was significant for both males and females after President Roh’s suicide as well as Ms. Choi’s, the impact of imitative effect was stronger for females after Ms. Choi’s suicide incident. This is because imitative suicide is characterised by the same gender as the celebrity[8,9,17]. In addition, females are more prone to have suicidal ideation than males in a similar distressed situation[29]. Furthermore, the incident had a big impact on depressed females in distress in Korea because of the symbolic power of the actress, along with her efforts to be a strong independent mother. For example, as soon as the relevant laws were revised, she became the first woman in Korea to change her children’s family name to that of her maiden family and the media story focusing on the positive aspects of the victim, actress Ms. Choi, could develop positive definitions of her suicide and facilitate suicidal behaviour of females[27].

The prominent increase in suicide deaths by the same suicide method during the Werther effect band strongly suggests the important role of media reporting in facilitating imitative suicides[8]. Our study showed that the method-specific suicide rate was not observed during the adjacent years of the suicide incident for both entertainment and politician celebrities. Indeed, the suicide methods of the two celebrities were reported without censorship, in the most prominent places in newspapers; this appears to have explicitly infringed on the guidelines for responsible reporting by media professionals that were devised with a view to preventing imitative suicide behaviours[30]. According to the guidelines, providing detailed information about the site and explicit description of the suicide method is to be avoided. Additionally, repetition of news reporting, particularly headlines, should be restricted; these rules should be considered especially in the reporting of celebrity suicides. 

The news articles examined in our study came from the three major newspapers in Korea, all of which mediate conservative views. However, if cable TV and Internet media are considered, the news on celebrity suicides is significantly magnified in Korea. In particular, the Internet is extensively prevalent, as Korea has the highest Internet access rate in the world[31]. In this regard, studies have reported that the stress and depression caused by reckless media reporting, including malicious comments in the mass media such as in newspapers and Internet news, have propelled celebrities to commit suicide in Korea[32,33]. The information technology infrastructure allows people to log on to the Internet or access media more or less anywhere and at any time[34]. Therefore, the general public can easily learn about the private lives of celebrities, and media reporting about celebrity suicide may now have an even greater diffusive effect on potential suicide among the general population with suicidal ideation or those who admire celebrities. 

At present, media-press guidelines have been developed by several countries to improve the quality of reporting, besides the World Health Organization (WHO) guideline[14,35]. Although suicide-related press guidelines exist in Korea, they are not enforceable. Thus, it appears an urgent matter to develop authoritative regulation to restrict indiscreet mass media reporting of suicides in Korea. For example, as suicide methods vary with age, it might be helpful to establish a suicide policy distinguished by age criteria, restricting access to harmful suicide methods and mass media such as Internet news as well as regulating media reporting on suicide-related news[17,36]. Furthermore, the task of educating the public about the suicide problem should be given to the mass media in close cooperation with public health workers to establish a more promising strategy, as recommended by the WHO[14,30,36,37]. 

Several limitations should be addressed in the interpretation of our findings. First, it is difficult to determine whether those who committed suicide following the recognised celebrity suicides had actually been exposed to the news. Second, although we controlled for various environmental factors in our analytical model, such as season, day of the week, average temperature, humidity, and unemployment rate, and the risks related to these factors seemed consistent with previous research, other factors might have influenced the suicide pattern, which could not be ruled out in this study[38,39]. Third, many celebrities have committed suicide over the past decade, as well as after the two suicide incidents considered in this study, but the cumulative effect of celebrity suicide on the imitative suicide risk has not been measured. Indeed, the ex-husband of Ms. Choi, who suffered from widespread reproach in the mass media, committed suicide in 2012. The cumulative and diffusive suicide risk as a result of celebrity suicides in Korea should be examined in future research. 

## Conclusions

The Werther effect was significantly apparent in Korea for 6 weeks and 4 weeks, respectively, after the suicides of entertainment celebrity Ms. Choi and former President Roh. The longer Werther effect band of entertainment celebrity could be explained by greater and positive frames of media coverage about her suicide. During the Werther effect periods following the death of both Ms. Choi and former President Roh, there was a significantly increased risk of suicide for all age groups, both genders, and the use of the same suicide method. This notably elevated suicide risk such as using specific suicide method during the same period of Werther effect band was not found for each of adjacent years of celebrities’ suicides. Considering the substantial impact of media coverage on both entertainment and politician celebrities, regulations and guidelines for the mass media should be urgently developed along with public efforts to enhance public mental health in Korea. 
